# Genetic Variation at Selected SNPs in the Leptin Gene and Association of Alleles with Markers of Kidney Disease in a Xhosa Population of South Africa

**DOI:** 10.1371/journal.pone.0009086

**Published:** 2010-02-05

**Authors:** Ikechi G. Okpechi, Brian L. Rayner, Lize van der Merwe, Bongani M. Mayosi, Adebowale Adeyemo, Nicki Tiffin, Rajkumar Ramesar

**Affiliations:** 1 Department of Medicine, Groote Schuur Hospital and University of Cape Town, Cape Town, South Africa; 2 Department of Statistics, University of the Western Cape, Cape Town, South Africa; 3 Centre for Research on Genomics and Global Health, National Human Genome Research Institute, Bethesda, Maryland, United States of America; 4 Division of Human Genetics, Institute for Infectious Diseases and Molecular Medicine, University of Cape Town, Cape Town, South Africa; VU University Medical Center and Center for Neurogenomics and Cognitive Research, VU University, Netherlands

## Abstract

**Background:**

Chronic kidney disease (CKD) is a significant public health problem that leads to end-stage renal disease (ESRD) with as many as 2 million people predicted to need therapy worldwide by 2010. Obesity is a risk factor for CKD and leptin, the obesity hormone, correlates with body fat mass and markers of renal function. A number of clinical and experimental studies have suggested a link between serum leptin and kidney disease. We hypothesised that variants in the *leptin* gene (*LEP*) may be associated with markers of CKD in indigenous black Africans.

**Methodology/Principal Findings:**

Black South Africans of Xhosa (distinct cultural Bantu-speaking population) descent were recruited for the study and four common polymorphisms of the *LEP* (rs7799039, rs791620, rs2167270 and STS-U43653 [ENSSNP5824596]) were analysed for genotype and haplotype association with urine albumin-to-creatinine ratio (UACR), estimated glomerular filtration rate (eGFR), Serum creatinine (Scr) and serum leptin level. In one of the four single nucleotide polymorphisms (SNPs) we examined, an association with the renal phenotypes was observed. Hypertensive subjects with the T allele (CT genotype) of the ENSSNP5824596 SNP had a significantly higher eGFR (p = 0.0141), and significantly lower Scr (p = 0.0137). This was confirmed by haplotype analysis. Also, the haplotype GAAC had a modest effect on urine albumin-to-creatinine ratio in normotensive subjects (p = 0.0482).

**Conclusions/Significance:**

These results suggest that genetic variations of the *LEP* may be associated with phenotypes that are markers of CKD in black Africans.

## Introduction

Chronic kidney disease (CKD) is a significant public health problem. CKD is irreversible and ultimately progresses to end-stage renal disease (ESRD), projected to affect 2 million people worldwide by 2010 worldwide [1], [2]. Microalbuminuria, a reversible and early measure of kidney disease marks the initiation of kidney disease and is a significant predictor of cardiovascular events and all-cause mortality in patients with diabetes, hypertension, and in the general population [3], [4]. Other measures of renal dysfunctions such as an increase in serum creatinine (Scr) and a reduced estimated glomerular filtration rate (eGFR) have also been shown to predict cardiovascular disease [5]. Estimates of the NHANES III dataset have shown that approximately 3 and 11% of the US population have abnormal Scr levels or microalbuminuria, respectively [6], [7].

Recently, obesity has been identified as a major driver for progressive kidney injury [8], [9]. A relative risk of 2.3 was reported of incident ESRD or kidney disease-related death in morbidly obese individuals who participated in the NHANES III survey [8]. Leptin is the obesity hormone synthesized mainly by white adipose tissue in humans [10] and its serum level shows strong correlation with body fat mass [11]. Mutations in the *leptin* gene (*LEP*) have been reported to cause severe obesity [10], [12] and may also contribute to the complications associated with obesity. The potential role of serum leptin in the pathogenesis of CKD has become increasingly recognised and a number of studies have demonstrated correlations between serum leptin and markers of renal function [13], [14]. Leptin stimulates the proliferation of cultured glomerular endothelial cells and induces mRNA expression and protein secretion of transforming growth factor-β1 (TGF-β1). Long-term infusion with leptin (3 weeks) has led to increased glomerular expression of type IV collagen [15]. Leptin has also been shown to stimulate synthesis of type I collagen in mesangial cells and type IV collagen in glomerular endothelial cells which contributes to extracellular matrix deposition, glomerulosclerosis, and proteinuria [16]. It is therefore possible that genetic variation/s in the *LEP*, possibly related to variation in serum leptin concentration may be associated with markers of kidney disease such as urine albumin-to-creatinine ratio (UACR), Scr and eGFR. We therefore hypothesized that polymorphisms of the *LEP* may have significant effects on markers of renal function in black Africans.

## Methods

**Figure 1:**
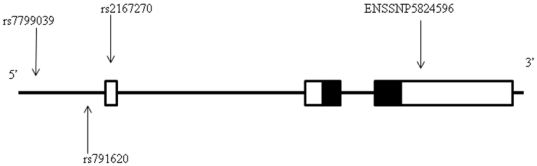
Position of four polymorphisms typed across the leptin (LEP) gene on chromosome 7. Dark shading indicates coding sequence.

The population sampling of this study, which is part of a larger study to determine the effects of obesity through the metabolic syndrome on kidney disease in an indigenous African population, was of a cross-sectional design and was carried out in Cape Town between May 2005 and July 2006. The study was approved by the joint Research Ethics Committee (REC) of the University of Cape Town and Groote Schuur Hospital. Written informed consent (approved by our REC) was obtained from each subject before they could enter the study. The method of recruitment has been previously described [14]. Briefly, two hundred and fifty three (253) ambulatory hypertensive subjects attending the Guguletu hypertension clinic and eighty-three (83) normotensive relatives in the community were recruited for the study. Although 336 subjects were recruited for the entire study, the sample sizes for the different single nucleotide polymorphisms (SNPs) that were examined differed and were fewer than that of the entire study population due to variation in the availability of high quality DNA, and incomplete successfully genotyping. We chose to study these non-coding polymorphisms because they capture the common haplotype variation across the *LEP* (figure 1) and also because they have been the commonly studied of the *LEP* SNPs in other populations, therefore providing a basis for comparison with our population.

The subjects were all of the same indigenous southern African tribal/cultural population group, namely of Xhosa origin, to ensure a homogenous population and to avoid confounding by population admixture which may lead to spurious results in gene association studies of unrelated individuals [17]. A questionnaire was administered to all participants to obtain relevant demographic information. Height, weight, waist and hip circumference were obtained. Body mass index was calculated from weight (kg) divided by height squared (m^2^). Blood pressure was measured in all the subjects using the same validated mercury sphygmomanometer. The average of 2 blood pressure measurements taken at least 2 minutes apart in the sitting position after about 5–10 minutes rest was recorded. Blood was drawn in the fasting state for routine chemistry including creatinine, lipids, glucose and for assay of leptin. Spot urine was also taken to measure the urine albumin-to-creatinine ratio (UACR). For all the tests, conventional assays were used in the chemical pathology laboratory on auto-analysers with appropriate quality control. The eGFR was calculated using the Modification of Diet in Renal Disease (MDRD) equation [18]:
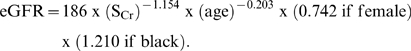



Serum leptin was measured using a commercially available human leptin radioimmunoassay kit (Linco Research, St. Charles, MO) with sensitivity 0.5 ng/ml, intra-assay precision 3.4–8.3%, and inter-assay precision 3.6–6.2%. All genetic analysis was carried out in the Division of Human Genetics at the University of Cape Town. Genomic DNA was isolated from peripheral blood lymphocytes using the Puregene DNA Isolation Kit (Gentra Systems, USA) according to the manufacturer's protocol. Polymerase Chain Reaction (PCR) was carried out individually for the four SNPs being tested. This was followed by restriction enzyme (RE) digest of the PCR products (table S1, S2, S3, S4). The primers (forward and reverse) used for the SNPs as well as the restriction enzymes used in the digest are shown in Table 1.

**Table 1:** Primers and the restriction enzymes used for genotyping.

SNP	Forward Primer	Reverse Primer	Restriction Enzyme
rs7799039	**5′** TTTCCTGTAATTTTCCCGTGAG **3′**	**5′** AAAGCAAAGACAGGCATAAAAA **3′**	HhaI
rs791620	**5′** CAACGAGGGCGCAGCCGTAT **3′**	**5′** AGTGTGCACCTCGCGGGGCCT **3′**	AscI
rs2167270	**5′** GCCCCGCGAGGTGCACACTG **3′**	**5′** GGGCCCTGTGGCCTGCCAAG **3′**	MspA1I
ENSSNP5824596	**5′** CGACCTGGAGAACCTCCG **3′**	**5′** GTCCTGGATAAGGGGTGT **3′**	HpyCH4IV

Phenotypes of interest had skewed distributions and were quantile normalised for analysis, consequently, only age and gender were adjusted for in the analysis. Untransformed values are summarised, for ease of interpretation. We had to use mixed-effects models for comparing the hypertensive to the normotensive groups, to enable us adjust for the relatedness between the normotensives and the hypertensives.

All genetic association analyses were stratified between hypertensive and normotensives, and adjusted for age and gender. We used linear models on quantile normalised traits. Genotypes were coded as categories (genotype; 2 degrees of freedom) and also as number of minor alleles (allelic; 1 degree of freedom). In cases where no minor allele homozygotes were observed, these analyses were equivalent. Haplotypes were imputed and analysed using an EM algorithm and generalised linear models [19]. We tested haplotype association with hypertension status, as well as for quantile normalised phenotypes stratified by hypertension diagnosis. We tested all possible haplotypes, from two to all four, from adjacent positions. R and R packages were used for statistical modelling; nlme for mixed-effects and haplo.stats for haplotype analyses [19] (R is a free software environment for statistical computing and graphics available from http://www.r-project.org).

## Results

The baseline features (demographic, clinical and biochemical) of all the participating subjects are summarised in table 2, stratified by diagnostic group. The median values of kidney disease phenotypes (Scr, eGFR and UACR) were not significantly different between the hypertensive and normotensive individuals. The genotype frequencies of the different polymorphisms are summarised in table 3 and agree closely with the genotype frequencies described at this polymorphism in Yoruba Africans in the Hapmap project [20]. All the typed SNPs were in Hardy-Weinberg equilibrium in the normotensive group (p<0.01), however, the SNP rs2167270 was not in Hardy-Weinberg equilibrium in the hypertensive subjects. No significant association was detected between hypertension status, obesity, the metabolic syndrome, hyperleptinaemia or gender and any of the 4 polymorphisms studied. In the hypertensive group, two phenotypes (Scr and eGFR), were associated with ENNSNP5824596, with the T allele significantly increasing eGFR (p = 0.0137) and decreasing Scr (p = 0.0186) (Table 4).

**Table 2:** Characteristics (median and interquartile range) of the study groups.

	Hypertensive				Normotensive				
	n	Median	LQ	UQ	n	Median	LQ	UQ	p-value
Age (yrs)	253	57.0	49.0	63.0	83	32.0	24.0	42.0	**<0.0001**
BMI (Kg/m^2^)	252	33.7	28.2	39.9	82	28.2	22.1	33.3	**0.0002**
SBP (mmHg)	252	151.5	137.0	165.3	83	122.0	114.0	138.0	**<0.0001**
DBP (mmHg)	252	94.0	87.0	102.0	83	82.0	76.0	89.0	**<0.0001**
FBG (mmol/L)	249	5.2	4.8	5.5	82	4.7	4.3	5.1	**0.0008**
TG (mmol/L)	252	1.2	0.8	1.7	83	0.8	0.6	1.1	**0.0292**
HDL-c (mmol/L)	252	1.4	1.1	1.6	83	1.5	1.3	1.9	**0.0027**
Leptin (ng/ml)	247	26.9	11.0	45.5	83	20.0	8.6	35.8	0.0986
Scr (µmol/L)	252	75.0	64.8	89.3	83	66.0	58.5	78.0	0.3067
eGFR(ml/min/1.73 m^2^)	252	99.0	85.8	114.0	78	125.0	109.3	143.8	0.1030
UACR (mg/mmol)	252	0.8	0.3	2.6	83	0.6	0.2	1.4	0.0606

P-values are for test of difference in quantile normalised characteristic between diagnostic groups, adjusted for age and gender and relatedness.

n = Number; Interquartile range is lower quartile (LQ) and upper quartile (UQ). BMI  =  body mass index; SBP  =  systolic blood pressure; DBP  =  diastolic blood pressure; FBG  =  fasting blood glucose; TG  =  triglyceride; HDL-c  =  high density lipoprotein cholesterol Scr  =  serum creatinine; eGFR  =  estimated glomerular filtration rate; UACR  =  urinary albumin-to-creatinine ratio.

**Table 3:** Genotype counts, allele counts and frequency distributions.

	Hypertensives		Normotensives		p-value
	n	f	n	f	
**rs7799039**					
Typed	191		59		
G	358	0.94	113	0.96	
A	24	0.06	5	0.04	0.1110
G/G	167	0.87	54	0.92	
G/A	24	0.13	5	0.08	0.1110
**rs791620**					
Typed	222		71		
C	404	0.91	132	0.93	
A	40	0.09	10	0.07	0.5873
C/C	187	0.84	61	0.86	
C/A	30	0.14	10	0.14	
A/A	5	0.02	0	0	NC
**rs2167270**					
Typed	219		74		
A	238	0.54	84	0.57	
G	200	0.46	64	0.43	0.8724
A/A	59	0.27	19	0.26	
A/G	120	0.55	46	0.62	
G/G	40	0.18	9	0.12	0.6912
**ENSSNP5824596**					
Typed	224		77		
C	417	0.93	144	0.94	
T	31	0.07	10	0.06	0.9286
C/C	193	0.86	68	0.88	
C/T	31	0.14	8	0.1	
T/T	0	0	1	0.01	NC

P-values are for test of difference in additive allelic and genotype distributions between diagnostic groups, adjusted for age and gender and relatedness.

n  =  count; f  =  frequency. NC  =  could not be calculated, because no minor allele homozygotes observed.

**Table 4:** Effect sizes (β) and p-values for genotype and allelic association with quantile normalised traits, adjusted for age and gender.

Hypertensives																
	rs7799039			rs791620					rs2167270					ENSSNP5824596		
	Genotype	Allelic		Genotype			Allelic		Genotype			Allelic		Genotype	Allelic	
	G/A	A		C/A	A/A		A		A/G	G/G		G		C/T	T	
	β	β	p-value	β	β	p-value	β	p-value	β	β	p-value	β	p-value	C/T	β	p-value
Leptin (ng/ml)	-	−0.02	0.9159	0.02	−0.16	0.8694	−0.02	0.8500	−0.12	−0.16	0.4814	−0.08	0.2492	-	−0.04	0.7923
Scr (µmol/L)	-	0.11	0.5206	−0.09	0.24	0.6943	0.00	0.9941	0.10	0.04	0.7038	0.03	0.7182	-	−0.37	**0.0186**
GFR(ml/min/1.73 m^2^)	-	−0.14	0.4428	0.02	−0.33	0.6804	−0.06	0.6529	−0.09	−0.04	0.7674	−0.03	0.7275	-	0.41	**0.0137**
uACR (mg/mmol)	-	−0.01	0.9680	−0.09	−0.70	0.2481	−0.20	0.1682	0.25	0.14	0.2228	0.09	0.3428	-	−0.04	0.8240

Normotensives																
Leptin (ng/ml)	-	−0.28	0.4670	-	-	-	−0.20	0.4603	−0.05	0.21	0.6711	0.07	0.6243	-	−0.27	0.2578
Scr (µmol/L)	-	−0.11	0.7760	-	-	-	−0.15	0.5833	−0.23	0.16	0.3230	0.02	0.8960	-	−0.26	0.2943
GFR(ml/min/1.73 m^2^)	-	0.25	0.5658	-	-	-	0.09	0.7813	0.37	0.02	0.2913	0.10	0.5857	-	0.36	0.2252
uACR (mg/mmol)	-	−0.10	0.8339	-	-	-	0.56	0.0970	−0.29	0.21	0.2957	0.03	0.8849	-	0.08	0.7863

Empty column means genotype result is exactly the same as for allele, because no minor allele homozygotes were observed.

Scr  =  Serum creatinine, eGFR  =  Estimated Glomerular filtration rate, UACR  =  Urine Albumin-to-creatinine ratio.

β (genotype) =  effect size (regression coefficient), estimated difference in transformed (quantile normalised) phenotype between individuals with a given genotype and individuals with the major allele homozygote genotype.

β (allelic)  =  effect size (regression coefficient), estimated difference in transformed (quantile normalised) phenotype for each additional allele.

The haplotypes GCGC and GCAC occurred more frequently than other haplotypes in the hypertensive and normotensive groups, respectively (Table 5). In the hypertensive group, haplotype GCAT yielded significantly higher values of eGFR than GCGC (p = 0.0278). The 3-way haplotype, excluding the first SNP, showed a similar pattern with CAT being associated with significantly higher values of eGFR than both CGC (p = 0.0255). In the last two polymorphisms, AT was also associated with significantly higher eGFR than GC (p = 0.0233) (Table 6).

**Table 5:** Inferred haplotype frequencies in the study population.

	Frequency	
Haplotype *	Hypertensive	Normotensive
GCAC	0.37	**0.46**
GCGC	**0.42**	0.36
GAAC	0.08	0.07
ACGC	0.04	0.05
GCAT	0.06	0.03
ACAC	0.03	0.00

*- Haplotypes are in their order on chromosome 7 (see Figure 1). Frequencies in bold characters denote the base (common) haplotypes.

**Table 6:** Results of test of association of haplotypes with markers of renal disease, adjusted for age and gender, in the hypertensive subjects.

rs7799039	rs791620	rs2167270	ENSSNP5824596	Scr		eGFR		UACR	
				β	p	β	p	β	p
A	C	A	C	0.29	0.3750	−0.41	0.1760	−0.03	0.9210
A	C	G	C	−0.12	0.7160	0.21	0.5210	−0.16	0.6490
G	A	A	C	0.00	0.9870	−0.06	0.7420	−0.29	0.1480
G	C	A	C	−0.04	0.7230	0.07	0.5340	−0.08	0.4970
G	C	A	T	−0.38	**0.0352**	0.42	**0.0278**	−0.12	0.5960
-	C	A	T	−0.38	**0.0318** †	0.42	**0.0255** †		
-	-	A	T	−0.36	**0.0293** ‡	0.39	**0.0233** ‡		
G	C	G	C	**Base haplotype**					

Tests are adjusted for age and sex.

β  =  estimated difference in transformed phenotype between individuals with a given haplotype and individuals with the base haplotype.

Scr  =  serum creatinine, eGFR  =  Estimated Glomerular filtration rate, UACR  =  Urine Albumin-to-creatinine ratio.

† - 3 –way haplotype analysis between CAT and CGC.

‡ - 2 – way haplotype analysis between AT and GC.

In the hypertensive group, haplotype GCAT yielded significantly lower values of Scr than GCGC (p = 0.0352). The 3-way haplotype, excluding the first SNP, showed a similar pattern with CAT being associated with significantly lower values of Scr than CGC (p = 0.0318) and in the last two polymorphisms, AT was associated with significantly lower Scr than GC (p = 0.0293) (Table 6).

In the normotensive group, the only significant association was between UACR and the 4-way haplotype. Urine albumin-to-creatinine ratio was significantly higher in GAAC than in GCAC (p = 0.0482) (Table 7). Linkage disequilibrium (LD) plot of the 4 *LEP* SNPs and a comparison with LD of the same region in HapMap YRI population which contains additional markers across the same span, revealed low pairwise r^2^ values indicating that LD across the region is weak (figure S1).

**Table 7:** Results of tests of association of haplotypes with markers of renal disease, adjusted for age and gender, in the normotensive subjects.

rs7799039	rs791620	rs2167270	ENSSNP5824596	Scr		eGFR		UACR	
				β	p	β	p	β	p
A	C	G	C	−0.11	0.7650	0.29	0.4920	0.18	0.6835
G	A	A	C	−0.21	0.4650	0.22	0.4930	0.71	**0.0482**
G	C	A	T	−0.28	0.4790	0.45	0.4820	1.35	0.0743
G	C	G	C	−0.01	0.9480	0.12	0.5380	0.19	0.3194
G	C	G	T	−0.34	0.4540	0.47	0.3000	−0.18	0.6845
G	C	A	C	**Base haplotype**					

β  =  estimated difference in transformed phenotype between individuals with a given haplotype and individuals with the base haplotype.

Scr  =  Serum creatinine, eGFR  =  Estimated Glomerular filtration rate, UACR  =  Urine Albumin-to-creatinine ratio.

## Discussion

We show from the results of this study that genetic variation in the *LEP* could have significant effects on renal disease phenotypes (markers of renal disease) in indigenous Africans. On the one hand, a marginal but significant effect was observed on microalbuminuria in normotensive subjects, while on the other hand a moderately significant and what is thought to be a “protective” effect was noticed with Scr and eGFR in hypertensive subjects. The reasons why this gene showed effects only on UACR in normotensives and then effects on Scr and eGFR alone in hypertensives are presently unclear. However, other factors associated with the hypertensive state may play a role. Although we consider that the effects of these polymorphisms on the phenotypes may be related to the renal actions of serum leptin, we have not shown that serum leptin has direct effects on the kidneys and can therefore not exclude autocrine and/or paracrine mediated actions of leptin in the kidney. The mechanism of action of serum leptin on the kidney has been previously described [15].

A number of studies have previously assessed the effects of the *LEP* on phenotypes of cardiovascular disease (including obesity) and cancer [21], [22], [23], [24], [25]. However, the results from many such studies have been largely inconsistent and difficult to replicate in other populations. For instance, Shintani et al [23] reported a positive association between a polymorphic tetranucleotide repeat (TTTC)_n_ polymorphism in the 3′ flanking region of the *LEP* and hypertension in a group of Japanese patients with essential hypertension. They found the frequency of the class I allele to be significantly higher in hypertensives compared with normotensive controls. In two other studies in South Americans and Italians in which the same polymorphism was examined, there was no association between the class I/II genotypes or alleles with hypertension or cardiovascular disease [24], [25].

The result of our study may be confirmatory of previous studies that have reported association between the common polymorphisms of the *LEP* and the various phenotypes they have assayed. One such confirmation relates to the “protective” effect of the T allele of the ENSSNP5824596 polymorphism from renal disease. To our knowledge, only one study [26] has reported on this “protective” effect from atherosclerosis in Caucasians. Gaukrodger et al demonstrated a significantly lower carotid intima medial thickness and pulse pressure in subjects with the T allele, compared to subjects without this allele (p = 0.0076 and p = 0.0001 respectively) [26]. Our study may have shown that this so-called “protective” association may exist in a different population using different phenotypes (Tables 4, 6 and 7).

This study was carried out on the assumption that common polymorphisms of the *LEP* may be associated with kidney disease given that serum leptin has been clinically and pathogenetically linked with markers of kidney disease [15], [16], [27]. As we know it, ESRD is common and more severe in people of African origin, although the exact reasons for this remain elusive. Differences in socio-economic status, higher prevalence of hypertension and an increased inherited susceptibility of indigenous Africans to kidney disease are all possible explanations [28], [29]. Additionally, as the prevalence of obesity continue to increase, its contribution to kidney disease globally and especially amongst the indigenous Africans cannot be ignored [30]. The overall median BMI of our study population was 32.5 kg/m^2^ (33.7 kg/m^2^ in the hypertensives and 28.2 kg/m^2^ in the normotensive group) (table 2).

This study is important in two ways: firstly, its focus on the relationship between the *LEP* and renal disease phenotypes. However, the value of this is diminished as the only significant effect we observed after multiple testing was the association of the T allele at ENNSNP5824596 among the hypertensives. This may have been due to the smaller sample size in the normotensive group or due to a possible conditional effect in these subjects with hypertension and increased BMI. Secondly, it may be important from the perspective of being carried out in an indigenous African population with no prior similar studies and in whom similar studies are generally under-represented. It therefore provides a prospect to evaluate the relationship between genetic polymorphisms and a specific complex disease (kidney disease) that is common amongst indigenous Africans. The study is however limited by its modest sample size with few SNPs studied and of being a cross-sectional type. A further and probably a more important limitation to it is our inability to show that these polymorphisms have any effect on tissue leptin, especially since we postulated that kidney disease in this population is associated with polymorphisms of the *LEP* through the renal effects of leptin. To demonstrate that these polymorphisms affect tissue (renal) leptin will require the design of further experimental studies. Finally, although modifiable risk factors for kidney disease are well known, a better understanding of obesity-related kidney disease will be necessary to control the progression of chronic renal disease to ESRD in black Africans.

## Supporting Information

Table S1 PCR assay of rs7799039
(0.04 MB DOC)

Table S2 PCR assay of rs791620
(0.03 MB DOC)

Table S3 PCR assay of rs2167270
(0.03 MB DOC)

Table S4 PCR assay of ENSSNP5824596
(0.03 MB DOC)

Figure S1 Linkage Disequilibrium (LD) plot visualized as a GOLD heat map
(1.49 MB TIF)
