# Supplementary material for: Genetic Variation at Selected SNPs in the Leptin Gene and Association of Alleles with Markers of Kidney Disease in a Xhosa Population of South Africa
Source: PLoS One. 2010 Feb 5;5(2):e9086. doi: 10.1371/journal.pone.0009086 (PMC2816711; doi:10.1371/journal.pone.0009086)
Supplement: Table S1 — PCR assay of rs7799039 (0.04 MB DOC) [file pone.0009086.s001.doc]

**Table S1:**

**PCR assay of rs7799039**

The assay involves producing a small fragment of 109bp with a cut at position 62 with the enzyme HhaI. This cut produces 2 fragments of 62bp and 47bp representing the homozygote genotype (GG). Due to the sizes of these products, only the 62bp fragment is often only seen on the 3% Agarose/Ethidium Bromide gel. Hence, only 1 band is seen in the homozygotes (GG) whereas 2 bands instead of 3 are seen in th heteroygotes (GA). HhaI cuts at 5’---G C G↓ C ----3’ or 3’---C↑ G C G ---5’.

**Amplicon:**

5’TTTCCTGTAATTTTCCCATGAGAACTATTCTTCTTTTGTTTTGTTTTGCGACAGGGTTGCGCTGATCCTCCCGCCTCAGTCTCCCTAAGTGCTGAGATGTTGCAGGAAG-3’

**PRIMERS**

**FP: 5’** TTTCCTGTAATTTTCCCGTGAG **3’** (22mer)

**RP:** **5’** AAAGCAAAGACAGGCATAAAAA **3’** (22mer)

**PROCEDURE:**

|  | Stock concentration | Volume (x1) | Final concentration |
| --- | --- | --- | --- |
| Distilled water |  | 16.9 |  |
| Buffer | 5x | 5.0 |  |
| dNTP | 5 μm | 1.0 | 1 μm |
| Forward primer | 100 nm/μL | 0.5 | 50 nm/assay |
| Reverse primer | 100 nm/μL | 0.5 | 50 nm/ assay |
| Pm Taq |  | 0.1 |  |
| DNA |  | 1.0 |  |

**PCR CONDITION:**

Stage 1: Denaturation:

94 ^o^C for 5 minutes (x 1 cycle)

Stage 2: Annealing:

94 ^o^C for 30 seconds (x 35 cycles)

48 ^o^C for 30 seconds (x 35 cycles)

72 ^o^C for 40 seconds (x 35 cycles)

Stage 3: Extension:

72 ^o^C for 7 minutes (x 1 cycle)

Restriction conditions: HhaI is incubated at 37 ^o^C for 4 hours using 0.5 μL of the restriction enzyme per 10.0 μL of PCR product.
