# Supplementary material for: Genetic Variation at Selected SNPs in the Leptin Gene and Association of Alleles with Markers of Kidney Disease in a Xhosa Population of South Africa
Source: PLoS One. 2010 Feb 5;5(2):e9086. doi: 10.1371/journal.pone.0009086 (PMC2816711; doi:10.1371/journal.pone.0009086)
Supplement: Table S2 — PCR assay of rs791620 (0.03 MB DOC) [file pone.0009086.s002.doc]

**Table S2:**

**PCR assay of rs791620**

Assaying for this SNP involves producing a fragment of 261bp with 1 cutting site for the restriction enzyme AscI. This cut at position 221 produces 2 fragments (221bp and 40bp) and represents the homozygote genotype (CC). The heterozygote (CA) has 3 bands representing 261bp, 221bp and 40 bp. The enzyme cuts at 5’---G G ↓C G C G C C---3’ or 3’---C C G C G C ↑G G ---5’.

**Amplicon (261)**

5’CAACGAGGGCGCAGCCGTATGCCCCAGCCCGCTCCGCGGAGCCCCTCACAGCCACCCCCGCCCCGACCGCGCCCCGCGGGCTCGAAGCACCTTCCCAAGGGGCTGGTCCTTGCGCCATAGTCGCGCCGGAGCCTCTGGAGGGACATCAAGGATTTCTCGCTCCTACCAGCCACCCCCAAATTTTTGGGAGGTACCCAAGGGTGCGCGCGTGGCTCCTGGCGCGCCGAGGCCCTCCCTCGAGGCCCCGCGAGGTGCACACT3’

**PRIMERS:**

**FP: 5’** CAACGAGGGCGCAGCCGTAT **3’** (20mer)

**RP:**  **5’** AGTGTGCACCTCGCGGGGCCT **3’** (20mer)

**PROCEDURE:**

|  | Stock concentration | Volume (x1) | Final concentration |
| --- | --- | --- | --- |
| Distilled water |  | 16.9 |  |
| Buffer | 5x | 5.0 |  |
| dNTP | 5 μm | 1.0 | 1 μm |
| Forward primer | 100 nm/μL | 0.5 | 50 nm/assay |
| Reverse primer | 100 nm/μL | 0.5 | 50 nm/ assay |
| Pm Taq |  | 0.1 |  |
| DNA |  | 1.0 |  |

**PCR CONDITIONS:**

Stage 1: Denaturation:

95 ^o^C for 5 minutes (x 1 cycle)

Stage 2: Annealing:

95 ^o^C for 30 seconds (x 35 cycles)

62 ^o^C for 30 seconds (x 35 cycles)

72 ^o^C for 40 seconds (x 35 cycles)

Stage 3: Extension:

72 ^o^C for 7 minutes (x 1 cycle)

Restriction conditions: AscI is incubated at 37 ^o^C for 4 hours using 0.5 μL of the restriction enzyme per 10.0 μL of PCR product.
