# Supplementary material for: Genetic Variation at Selected SNPs in the Leptin Gene and Association of Alleles with Markers of Kidney Disease in a Xhosa Population of South Africa
Source: PLoS One. 2010 Feb 5;5(2):e9086. doi: 10.1371/journal.pone.0009086 (PMC2816711; doi:10.1371/journal.pone.0009086)
Supplement: Table S3 — PCR assay of rs2167270 (0.03 MB DOC) [file pone.0009086.s003.doc]

**Table S3:**

**PCR assay of rs2167270**

The assay involves producing a fragment of 258 bp with 1 cutting site at position 186 of the fragment for the restriction enzyme MspA1I (cuts at 5’---C(A/C)G↓C(G/T)G---3’ or 3’---G(T/G)C↑G(C/A)C---5’). A cut at this position (homozygote GG) will result in 2 bands (186 bp and 62 bp fragments). Heterozygotes thus display 3 bands: 258 bp, 186 bp and 62 bp fragments.

**AMPLICON (258)**

**5’**GCCCCGCGAGGTGCACACTGCGGGCCCAGGGCTAGCAGCCGCCCGGCACGTCGCTACCCTGAGGGGCGGGGCGGGAGCTGGCGCTAGAAATGCGCCGGGGCCTGCGGGGCAGTTGCGCAAGTTGTGATCGGGCCGCTATAAGAGGGGCGGGCAGGCATGGAGCCCCGTAGGAATCGCAGCGCCAGCGGTTGCAAGGTAAGGCCCCGGCGCGCTCCTTCCTCCTTCTCTGCTGGTCTTTCTTGGCAG GCCACAGGGC CC-3’

**PRIMERS:**

**FP:** **5’-** GCCCCGCGAGGTGCACACTG -**3’** (20mer)

**RP:** **5’-** GGGCCCTGTGGCCTGCCAAG -**3’** (20mer)

**PROCEDURE:**

|  | Stock concentration | Volume (μL) | Final concentration |
| --- | --- | --- | --- |
| Distilled water |  | 16.9 |  |
| Buffer | 5x | 5.0 |  |
| dNTP | 5 μm | 1.0 | 1 μm |
| Forward primer | 100 nm/μL | 0.5 | 50 nm/assay |
| Reverse primer | 100 nm/μL | 0.5 | 50 nm/ assay |
| Pm Taq |  | 0.1 |  |
| DNA |  | 1.0 |  |

**PCR CONDITIONS:**

Stage 1: Denaturation:

94 ^o^C for 5 minutes (x 1 cycle)

Stage 2: Annealing:

94 ^o^C for 30 seconds (x 35 cycles)

62 ^o^C for 30 seconds (x 35 cycles)

72 ^o^C for 40 seconds (x 35 cycles)

Stage 3: Extension:

72 ^o^C for 7 minutes (x 1 cycle)

Restriction conditions: MspA1I is incubated at 37 ^o^C for 4 hours using 0.5 μL of the restriction enzyme per 1.5 μL of PCR product
