# Supplementary material for: Genetic Variation at Selected SNPs in the Leptin Gene and Association of Alleles with Markers of Kidney Disease in a Xhosa Population of South Africa
Source: PLoS One. 2010 Feb 5;5(2):e9086. doi: 10.1371/journal.pone.0009086 (PMC2816711; doi:10.1371/journal.pone.0009086)
Supplement: Table S4 — PCR assay of ENSSNP5824596 (0.03 MB DOC) [file pone.0009086.s004.doc]

**Table S4:**

**PCR assay of ENSSNP5824596**

The assay involves producing a fragment of 316 bp with 2 cutting sites at positions 32 and 245 of the fragment for the restriction enzyme TaiII (cuts at 5----A C G T↓----3 or 3----↑T G C A----5). The homozygote genotype (CC) thus has 3 bands: 213bp, 71bp and 32bp fragments, while the heterozygote (CT) has 4 bands: 284bp, 213bp, 71bp and 32bp fragments.

**AMPLICON (316)**

5’CGACCTGGAGAACCTCCGGGATCTTCTTCACGTGCTGGCCTTCTCTAAGAGCTGCCACTTGCCCTGGGCCAGTGGCCTGGAGACCTTGGACAGCCTGGGGGGTGTCCTGGAAGCTTCAGGCTACTCCACAGAGGTGGTGGCCCTGAGCAGGCTGCAGGGGTCTCTGCAGGACATGCTGTGGCAGCTGGACCTCAGCCCTGGGTGC**TGA**GGCCTTGAAGGTCACTCTTCCTGCAAGGACTACGTTAAGGGAAGGAACTCTGGCTTCCAGGTATCTCCAGGATTGAAGAGCATT GCATGGACAC CCCTTATCCA GGAC-3’

**PRIMERS:**

**FP:**  **5’-** CGACCTGGAGAACCTCCG- **3**‘(18mer)

**RP: 5’** -GTCCTGGATAAGGGGTGT- **3’** (18mer)

**PROCEDURE:**

|  | Stock concentration | Volume (x1) | Final concentration |
| --- | --- | --- | --- |
| Distilled water |  | 16.9 |  |
| Buffer | 5x | 5.0 |  |
| dNTP | 5 μm | 1.0 | 1 μm |
| Forward primer | 100 nm/μL | 0.5 | 50 nm/assay |
| Reverse primer | 100 nm/μL | 0.5 | 50 nm/ assay |
| Pm Taq |  | 0.1 |  |
| DNA |  | 1.0 |  |

**PCR CONDITIONS:**

Stage 1: Denaturation:

94 ^o^C for 5 minutes (x 1 cycle)

Stage 2: Annealing:

94 ^o^C for 30 seconds (x 35 cycles)

53 ^o^C for 30 seconds (x 35 cycles)

72 ^o^C for 40 seconds (x 35 cycles)

Stage 3: Extension:

72 ^o^C for 7 minutes (x 1 cycle)

Restriction conditions: TaiI (MaeII) is incubated at 65 ^o^C for 4 hours using 0.5 μL of the restriction enzyme per 10.0μL of PCR product.
