# Supplementary figures and images for: Genetic Variation at Selected SNPs in the Leptin Gene and Association of Alleles with Markers of Kidney Disease in a Xhosa Population of South Africa
Source: PLoS One. 2010 Feb 5;5(2):e9086. doi: 10.1371/journal.pone.0009086 (PMC2816711; doi:10.1371/journal.pone.0009086)

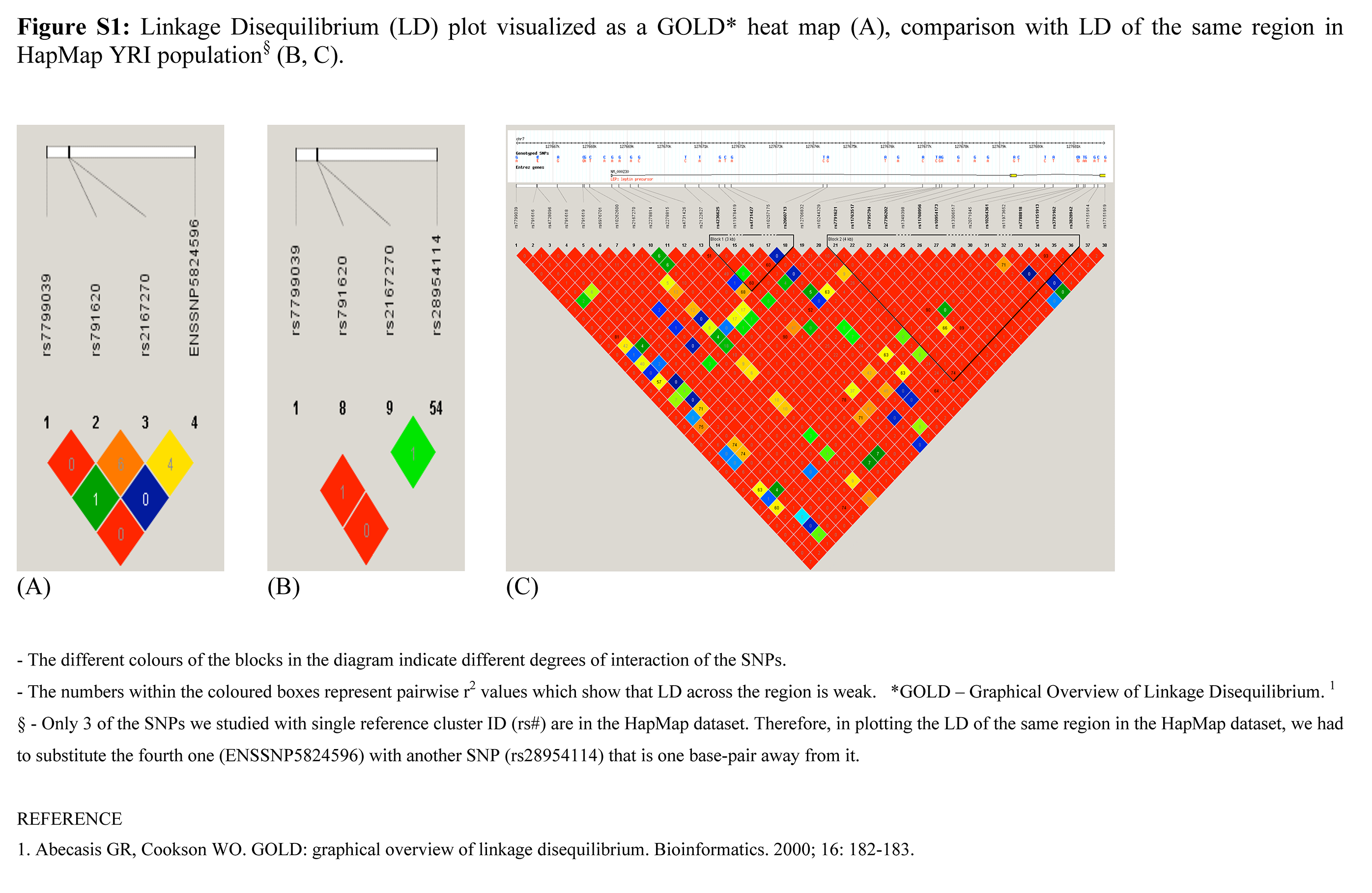

Supplement: Figure S1 — Linkage Disequilibrium (LD) plot visualized as a GOLD heat map (1.49 MB TIF) [file pone.0009086.s005.tif]
